# Intraperitoneal and Extraperitoneal Colonic Perforation Following Diagnostic and Therapeutic Colonoscopy with Crohn’s-related Stricture Dilation

**DOI:** 10.7759/cureus.7162

**Published:** 2020-03-02

**Authors:** Emily Weng, Damian N Valencia, Zoltan A Krudy, Median Ali

**Affiliations:** 1 Internal Medicine, Kettering Medical Center, Kettering, USA; 2 Internal Medicine, Kettering Medical Center, Dayton, USA; 3 Pulmonary Medicine and Critical Care, Kettering Medical Center, Kettering, USA

**Keywords:** stricture, perforation, colonoscopy, intraperitoneal, extraperitoneal, iatrogenic, pneumoperitoneum, pneumothorax, chron's

## Abstract

Colonic perforation is an uncommon but known and feared complication of colonoscopy, which carries a high mortality rate. We present an uncommon case of extensive intra- and extraperitoneal air following colonic perforation in a patient undergoing inpatient colonoscopy for evaluation of unintentional weight loss and constipation. During colonoscopy, a splenic flexure stricture was identified and dilated. Postprocedural hemodynamic instability prompted further imaging which revealed pneumoperitoneum, bilateral pneumothorax, pneumomediastinum, pneumopericardium, and severe subcutaneous emphysema. Emergent exploratory laparotomy found perforation of the proximal transverse colon which required resection and transverse colostomy placement. The patient also underwent bilateral chest tube placement and was treated with antibiotics for peritonitis. The patient was eventually diagnosed with Crohn’s disease and discharged to an extended care facility with outpatient follow-up. Extraperitoneal colonic perforations are fairly rare, and to our knowledge, we present the most severe case that has been published in recent years.

## Introduction

Iatrogenic colonic perforation from colonoscopy is uncommon with an overall complication rate of 0.01%-0.3%, with 3% of cases related to endoscopic balloon dilation of Crohn’s disease-related strictures [1-3]. Overall morbidity and mortality rates have been reported as high as 48.7% and 25.6%, respectively [4]. Perforations are typically the result of mechanical trauma, barotrauma, or electrocautery injury, and they more commonly occur during therapeutic colonoscopies. Both plain abdominal radiograph and computed tomography (CT) of the abdomen are used to identify free subdiaphragmatic air, although CT has been proven superior [1,5]. The majority of perforations are intraperitoneal. To date of this publication, only 31 cases of extraperitoneal perforation and 21 cases of concomitant intra- and extraperitoneal perforation have been reported worldwide [6].

Since 2017, there have been few reports detailing pathological colonic perforation, with only three describing extraperitoneal diverticular perforation [7,8]. Only one case reported pneumothorax and three extraperitoneal perforation after diagnostic colonoscopy. These cases were treated with conservative therapy of bowel rest, total parenteric nutrition (TPN), endoscopic repair, or laparotomy with anastomosis [9-12]. Other extraperitoneal manifestations, such as subcutaneous emphysema and mediastinal air, result from communication of fascial planes, mostly near large vessels that connect with cervical soft tissue between the mediastinum and retroperitoneum [6,13]. We report a case of extensive intra- and extraperitoneal colonic perforation following a Crohn’s disease-related stricture dilation.

## Case presentation

The patient is a 39-year-old female with past medical history of tobacco use (7.5 pack-years), anxiety, and depression. She was not on any home medications. The patient presented with three months of anorexia, malaise, weakness, and fatigue, associated with unintentional weight loss (20-30 lbs). The patient also reported several weeks of diarrhea followed by constipation, and a dull but intermittently sharp pain in her left lower and upper abdominal quadrants. The pain was aggravated by pressure and with any oral intake; alleviating factors included heat and bowel rest. The patient was also recently admitted (two months prior) for similar symptoms, but left against medical advice after she was unable to tolerate bowel preparation for colonoscopy.

On initial presentation to the emergency department, the patient was hemodynamically stable. Blood pressure was 108/84 mmHg, oxygen saturation was 98% on room air, and heart rate was 103 beats per minute. The patient was cachectic and appeared much older than her stated age. Physical examination was significant for angular cheilitis and white plaques on an erythematous oral mucosa. Significant dental decay was noted with numerous dental caries and a maxillary denture. Her abdomen was tender to palpation in the left abdominal quadrants, left flank, and left anterolateral chest wall. Mottled skin, consistent with a heating pad burn, was observed along the left lateral abdomen.

Initial serologic evaluation was significant for leukocytosis (white cell count 15.8 K/uL), anemia (hemoglobin 11.7 g/dL), thrombocytosis (platelets 453 K/uL), hypokalemia (potassium 2.5 mmol/L), hypoalbuminemia (albumin 2.7 g/dL), elevated C-reactive protein (9.4 mg/L), and an elevated erythrocyte sedimentation rate (37 mm/h). Urinalysis was positive for hyaline casts (10-20/lpf) and a small amount of bilirubin. CT of the abdomen and pelvis revealed colonic wall thickening at the splenic flexure and proximal descending colon with some adjacent small volume fluid.

At this point in time, the patient was started on piperacillin-tazobactam (Zosyn) 3.375 mg intravenously three times daily for suspected bacterial colitis and admitted for further gastroenterologic evaluation. Colonoscopy revealed a descending colon and splenic flexure stricture approximately 45 cm from the rectum without mass lesions. Dilation to 13.5 mm via a through-the-scope colonic balloon dilator was performed. Retained partially obstructing fecal matter was removed, and the scope was advanced into the transverse colon. During the colonoscopy, the patient's abdomen became progressively more distended and then developed crepitus in the chest wall and neck. Colonoscopy was aborted after subcutaneous air was noted in the abdominal wall. Upon arrival to the intensive care unit, the patient was noted to have diffuse crepitus above the diaphragm, throughout the chest wall, neck, and right eye. The patient was intubated, sedated and mechanically ventilated for respiratory distress. Care was assumed by the pulmonary medicine and critical care team.

**Figure 1:**
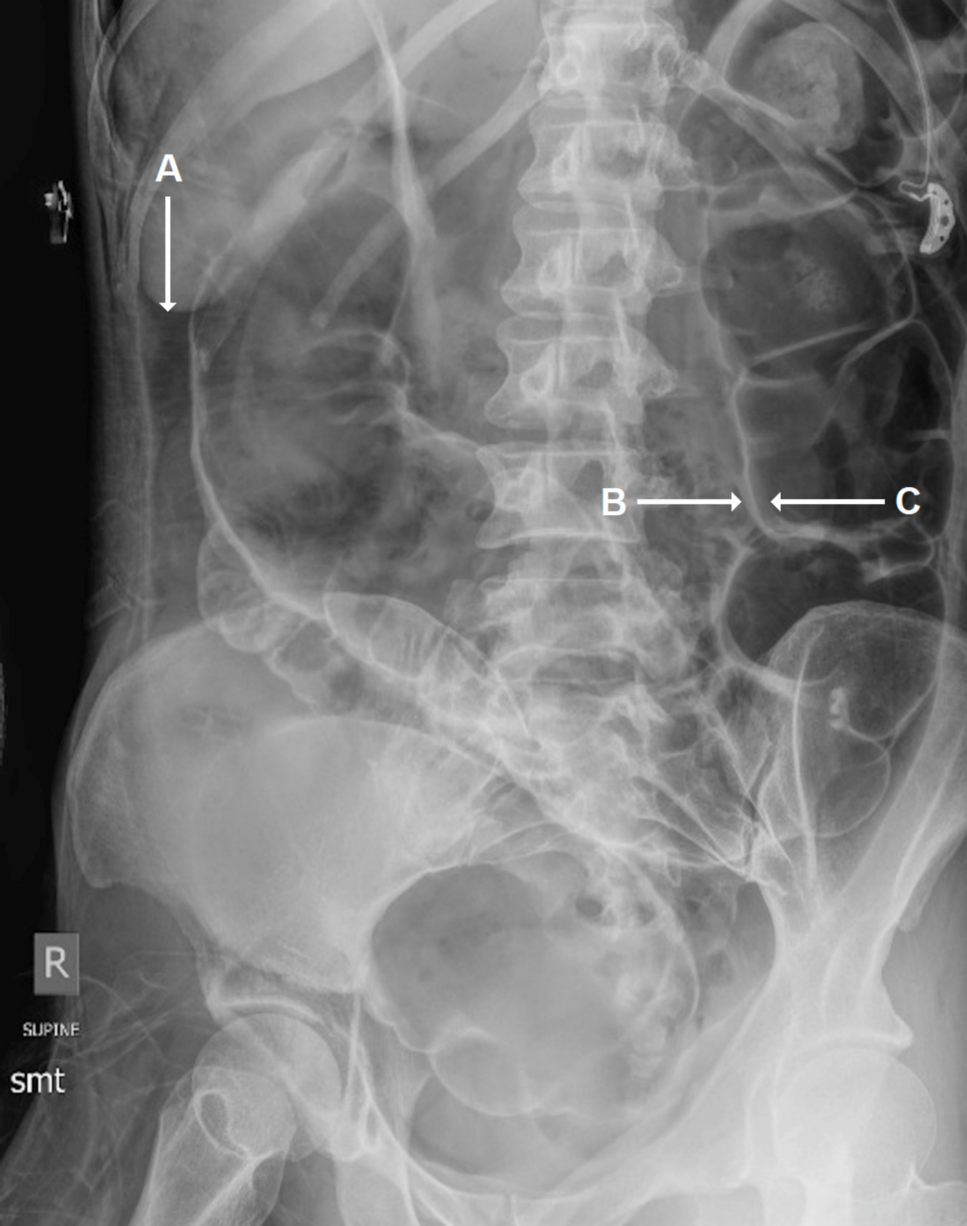
Abdominal Radiograph Abdominal radiograph depicting intraperitoneal air (A) and both peritoneal (B) and luminal (C) colonic wall air (Rigler’s or double wall sign).

Abdominal radiograph revealed significant intraperitoneal air and was positive for Rigler's sign, consistent with pneumoperitoneum (Figure 1).

**Figure 2:**
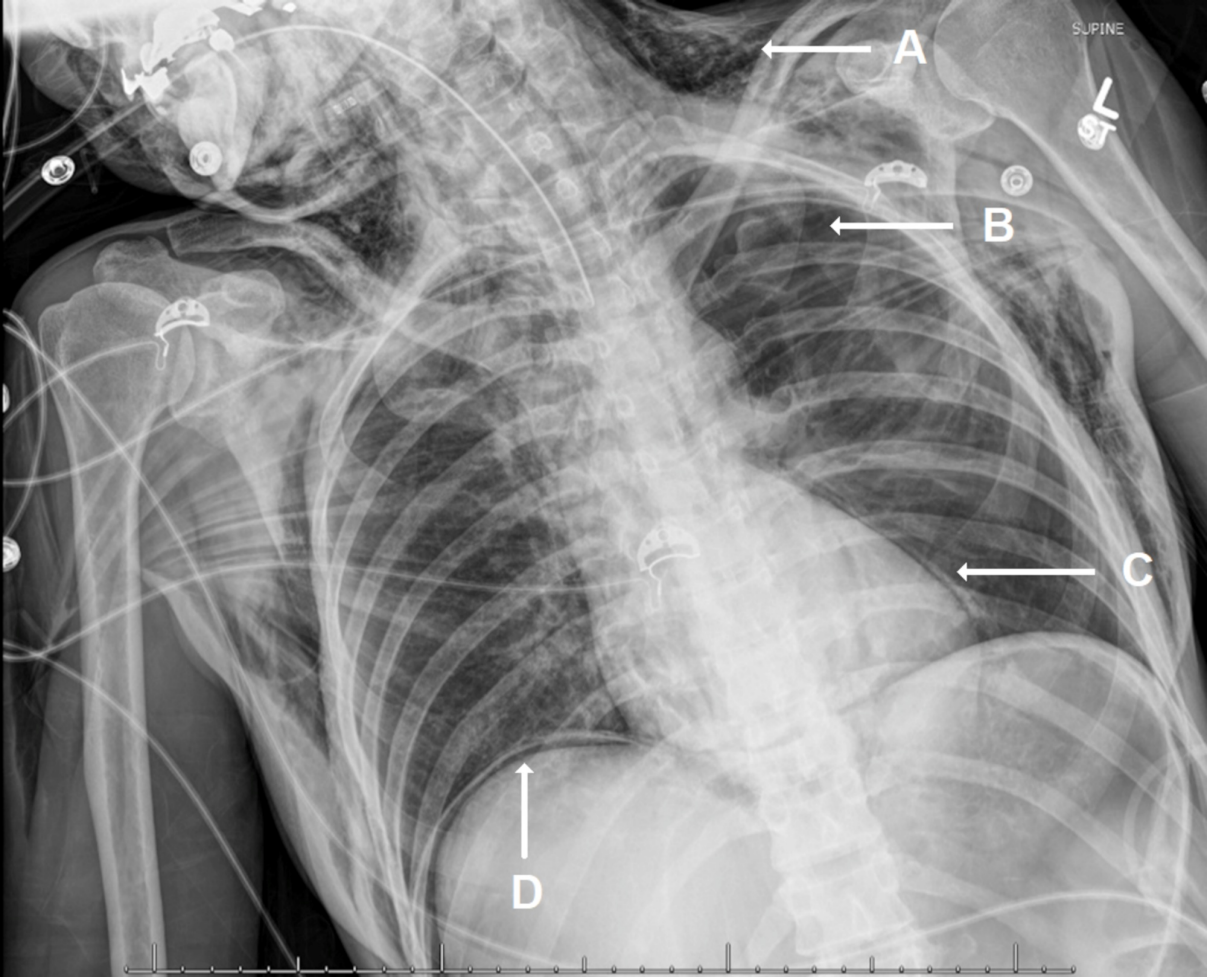
Chest Radiograph Anterior-posterior chest radiograph depicting diffuse subcutaneous emphysema (A), pneumothorax (B), pneumomediastinum (C), and subdiaphragmatic air (D) (diaphragm sign).

Chest radiograph revealed extensive subcutaneous emphysema, bilateral pneumothorax, and pneumomediastinum, and was positive for diaphragm sign (Figure 2). 

**Figure 3:**
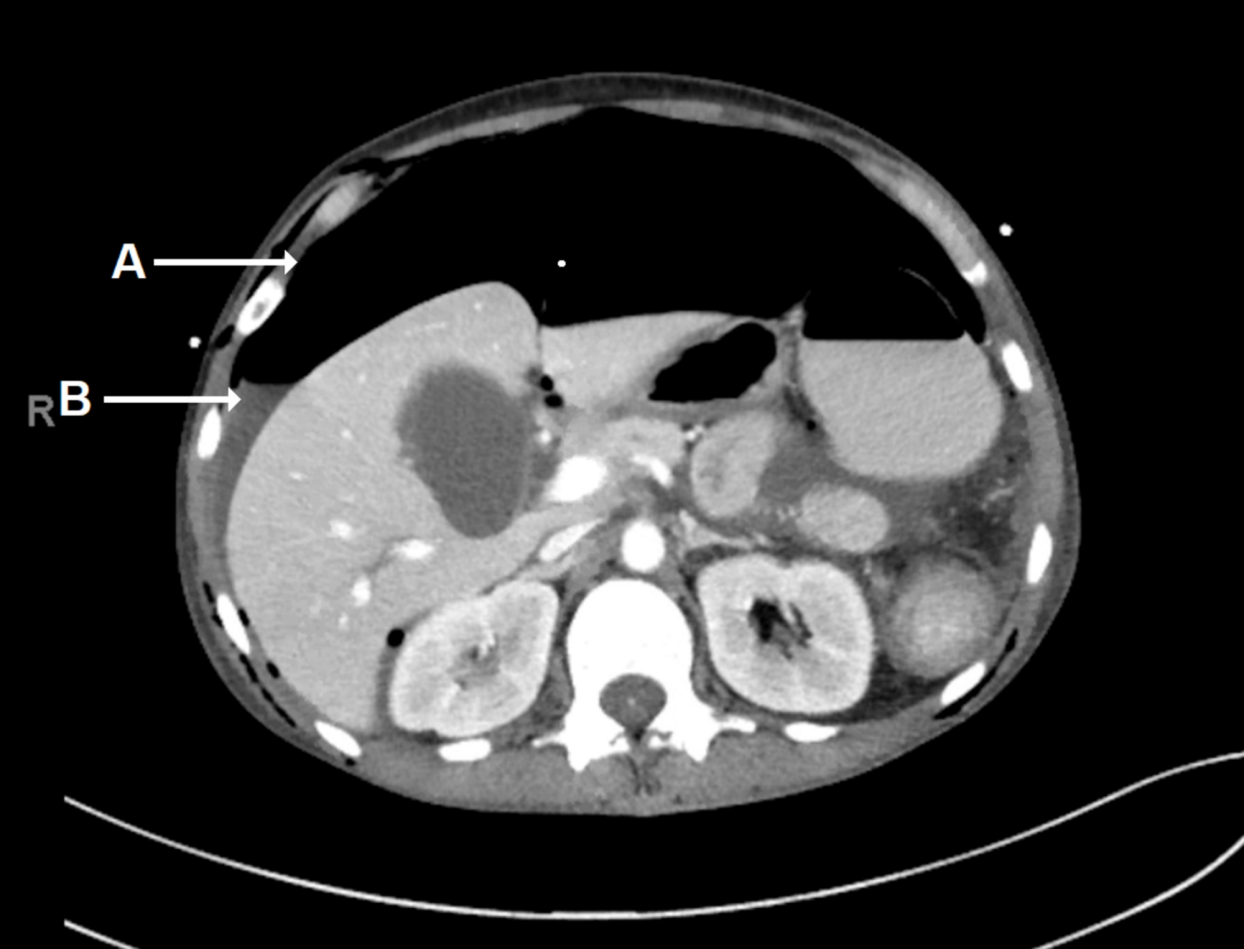
CT of the Abdomen/Pelvis CT of the abdomen/pelvis with intravenous contrast only, in the axial plane, demonstrating pneumoperitoneum (A) with small-to-moderate volume ascites (B).

CT of the abdomen and pelvis showed significant pneumoperitoneum and ascites, consistent with the suspected colonic perforation (Figure 3).

**Figure 4:**
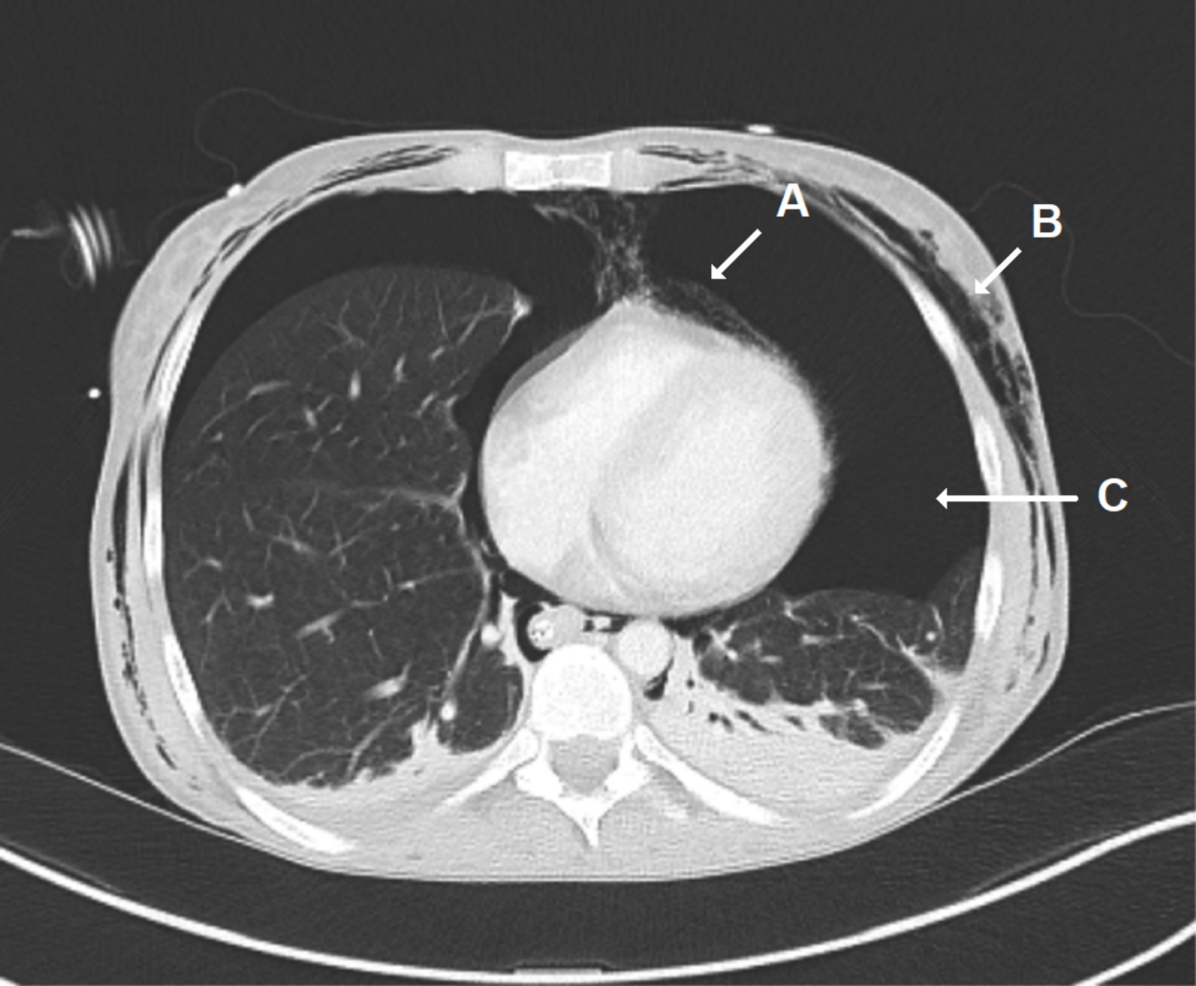
CT of the Chest CT of the chest with contrast, in the axial plane, revealing pneumomediastinum (A), diffuse subcutaneous chest wall emphysema (B), and pneumothorax (C).

CT of the chest again confirmed pneumomediastinum, subcutaneous emphysema, and bilateral pneumothorax (Figure 4). 

The patient underwent bilateral chest tube placement and was emergently taken to the operating room for exploratory laparotomy. A perforation at the proximal transverse colon with stool in the peritoneum was identified, along with a splenic flexure mass and a serosal tear at the cecum without perforation. A left hemicolectomy with mobilization of the colon at the splenic flexure was performed. The abdomen was temporarily closed with a surgical wound vacuum. On postoperative day 2, the patient returned to the operating room for a right hemicolectomy with ileocolic anastomosis, diverting loop ileostomy, Jackson-Pratt (JP) drain placement, and fascial closure. The patient was extubated, and chest tubes were removed on postoperative day 3. Pathology was suggestive of atypical Crohn’s disease without small intestine involvement.

The patient was started on TPN via peripherally inserted central catheter from postoperative days 3-6 while awaiting initiation of oral intake. After tolerating enteral nutrition, the patient had sufficient ostomy output and was discharged in stable condition to an extended care facility with a midline abdominal wound vacuum and right lower quadrant JP drain, both of which were removed in office one week after discharge. After the 15-day admission, the patient was discharged with no new medications. Plans were made to begin anti-tumor necrosis factor therapy six to eight weeks after surgery, with repeat endoscopic evaluation in six to twelve months after ileostomy reversal. Unfortunately, the patient was readmitted four days later for suspected small bowel obstruction, which was treated medically with bowel rest and small bowel decompression (nasogastric tube placement to low intermittent wall suction). The patient was advised to follow up with primary care and gastroenterology prior to discussion of ileostomy reversal with general surgery.

## Discussion

Although iatrogenic colonic perforation due to colonoscopy has low incidence, perforation can have catastrophic consequences with up to 25% mortality [14]. The rate of colonic perforation is higher during therapeutic colonoscopy compared to diagnostic colonoscopy, with incidences estimated at 0.02%-8.0% and 0.016%-0.8%, respectively [15]. The sigmoid colon is a common location for diverticula and polyps, which may lead to mucosal weakening and may be the cause of increased perforation rates when compared to other sites in the colon. The cecum is also a common site for perforation, most often due to barotrauma, owing to its thinner muscular layer and larger diameter [6,13]. Perforations can be intraperitoneal, extraperitoneal, or both. Extraperitoneal colonic perforations are fairly rare, and to our knowledge, we present the most severe case that has been published in recent years [16]. Unlike intraperitoneal perforations which can manifest as peritonitis, retroperitoneal perforations can be difficult to diagnose clinically as symptoms can be atypical [17]. During intraprocedural insufflation, patients often complain of abdominal cramping, further complicating the diagnosis of peritonitis. Given the potentially life-threatening complication of colonic perforation, the index of suspicion should remain high in the presence of other postprocedural symptoms including respiratory distress and crepitus along the chest wall, neck, or hips.

The case we present was complicated by underlying Crohn’s disease. Fibrostenosing Crohn’s disease is not uncommon, with diagnosis often coinciding with decline in quality of life. Patients often present with complaints of postprandial abdominal pain, nausea, vomiting, bloating, and weight loss, much like the case detailed above [18]. Approximately 75% of patients will eventually undergo surgical intervention at least once in their lifetime [19]. Risk factors for perforation include strictures greater than 5 cm, multiple strictures, inflammatory stricture without medical optimization, strictures caused by extrinsic compression, fistulization within 5 cm of area to be dilated, adjacent perforation or intra-abdominal collection, complete duodenal stricture, tortuous or tethered small bowel, or significant stricture angulation [18]. Endoscopic balloon dilatation appears to be safe, with serious complication rate at 3% or less [20].

Not all colonic perforations require surgical management. There have been case reports of colonic perforations of lesser magnitude managed conservatively with bowel rest and antibiotics. There have also been studies supporting endoscopic repair such as endoscopic clipping or suturing closure [14]. However, in the case we present, the patient's condition was unstable, and the complications of colonic perforation were so extensive that surgical intervention was necessary.

## Conclusions

We present a rare case of colonic perforation from endoscopic balloon dilation of a Crohn’s disease-related stricture at the splenic flexure, with extensive intra- and extraperitoneal manifestations requiring emergent surgical intervention. Although uncommon, colonic perforation carries a high mortality and early recognition is imperative. We hope this case emphasizes the importance of atypical symptom recognition, early diagnosis, and prompt treatment of iatrogenic colonic perforation from colonoscopy.
